# Pediatric Obturator Internus Muscle Myxoma

**DOI:** 10.5435/JAAOSGlobal-D-20-00099

**Published:** 2021-01-19

**Authors:** Ehab S. Saleh

**Affiliations:** From the Section of Pediatric Orthopedic Surgery, William Beaumont Hospital, Royal Oak, MI, and the Department of Orthopedic Surgery, Oakland University William Beaumont School of Medicine, Rochester, MI.

## Abstract

Intramuscular myxoma is a rare benign tumor that presents as a slow-growing, deeply seated mass confined within a skeletal muscle. Histologically, these lesions most resemble umbilical cord tissue. They mostly occur in people between 40 and 70 years old, with a 57% female predilection. These tumors are very rare in children. Only one pediatric intramuscular myxoma case is reported in the literature. The goal of this study is to report the case of a 13-year-old girl who presented to our hospital emergency department in 2018 with right hip pain, elevated inflammatory markers, and fever; her initial differential diagnosis was hip septic arthritis, pelvic osteomyelitis, and pelvic abscess. A pelvic MRI revealed a well-defined enhancing round lesion in the right obturator internus muscle. The diagnosis was conformed with a CT-guided core biopsy. The patient's symptoms improved with conservative management, and she continues to be doing well 2 years later. Pediatric pelvic intramuscular myxomas are extremely rare; however, they can have a presentation that mimic a more serious condition such as hip septic arthritis, pelvic osteomyelitis, and pelvic abscess and should be considered in the differential diagnosis in a pediatric patient presenting with hip pain.

In 1948, Purdy Stout defined myxomas as a true neoplasm composed of stellate cells set in a loose mucoid stroma through which course very delicate reticulin fibers in various directions, resembling primitive mesenchyme.^1,2,3,4^ Using this definition, he studied 49 cases of myxoma at various anatomic locations, reported at Columbia University, and reviewed the literature to find 95 more cases, all of them were in the heart.

In 1965, Enzinger^5^ recognized intramuscular myxoma as a distinct entity. From a group of 200 myxomas from various sites, 34 intramuscular myxomas (17%) were identified in his study.

In a study that was done in 2002, intramuscular myxoma was described as rare in young people, and until that year, it has not been reported in children.^3^ Since then, pediatric intramuscular myxoma has been reported in a 22-month-old girl with a myxoma in the posterior cervical triangle.^1^

Intramuscular myxoma involving the obturator internus muscle has not been reported before, although there are reports of obturator externus muscle intramuscular myxoma.^6^

In this report, the case of a 13-year-old girl with a right obturator internus intramuscular myxoma is described, with a review of the literature.

## Case Report

A 13-year-old girl presented to the emergency department with a 3-day history of right hip pain, associated with intermittent fever; the pain progressed to the point where she was not able to bear weight on the right lower extremity; her white blood cell, erythrocyte sedimentation rate, and C-reactive protein were elevated; rheumatological evaluation was negative, except for a positive antinuclear antibody titer of 1:160. She also reported right shoulder pain that increased with motion.

**Figure 1:**
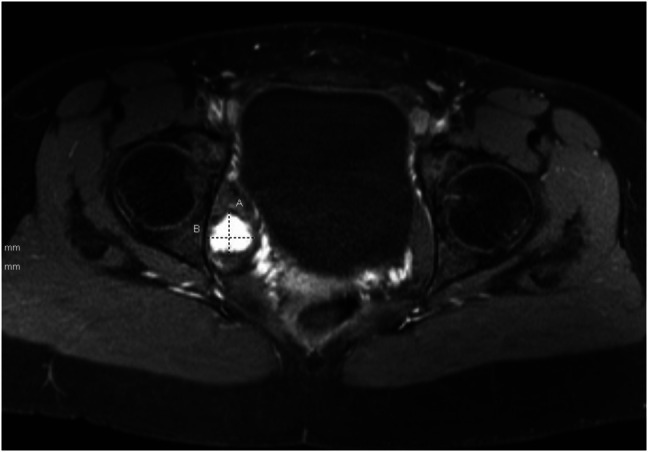
Axial MRI view showing the right obturator internus intramuscular myxoma.

**Figure 2:**
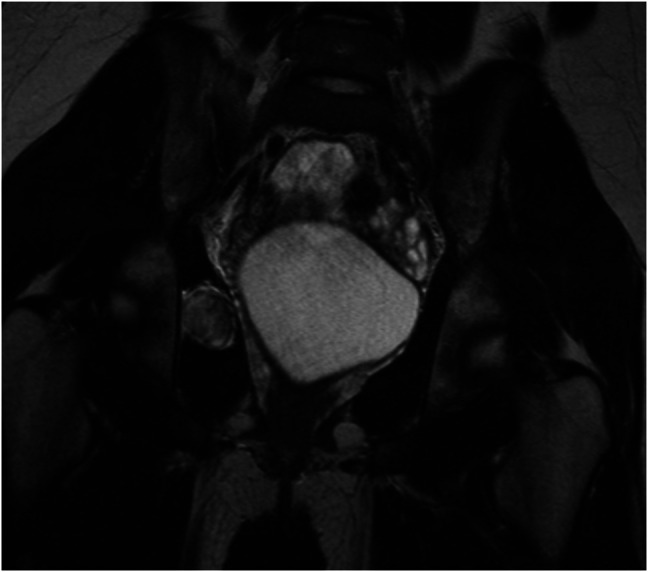
Coronal MRI view showing the right obturator internus intramuscular myxoma.

She was evaluated with a hip radiograph and ultrasonography, which were normal, followed by a pelvic MRI scan with contrast, which did show a well-defined enhancing round lesion in the right obturator internus muscle without associated surrounding edema or enhancement, with a rim of fat seen at the periphery of the lesion. In addition, nonspecific edema was noted involving the symphysis pubis and the right sacroiliac joint (Figures 1 and 2). The differential diagnosis given by the radiologist for the mass included a neurogenic or myxoid neoplasm.

She was also evaluated with a right shoulder MRI, which was positive for nonspecific mild amount of fluid and enhancement in the subacromial, subdeltoid bursa, and the rotator cuff interval, and tendinopathy of the supraspinatus tendon. Our clinical diagnosis for the shoulder was subacromial bursitis.

The pediatric infectious disease team started the patient on empiric IV vancomycin. They also recommended an echocardiogram to assess for atrial myxoma; the echo was normal.

Our interventional radiologist was consulted; the best approach for a CT-guided biopsy was discussed with him and with the orthopaedic oncologist. It was decided that a posterior approach would be the best in this case.

Under general anesthesia, the patient was positioned in the prone position and the overlying skin was prepped and draped in a sterile fashion. The subcutaneous tissues were infiltrated with 1% lidocaine for local anesthesia. Using CT guidance, a 17-gauge introducer was advanced to the lesion, which was found to be solid, and through this, five, 18-gauge core biopsy samples were obtained. The specimens were sent for pathological examination and for culture. The pathology result was positive for myxoma. The cultures were negative.

The pediatric infectious disease team discontinued the vancomycin on day 5 once all culture results were confirmed to be negative.

The patient's course during hospitalization was of gradual clinical improvement, with supportive treatment that included NSAID and mobilization by the therapist. She was gradually able to ambulate with a walker and her right shoulder pain gradually improved. Based on her clinical improvement, and after a discussion with the family, a decision was made to manage the right obturator internus intramuscular myxoma with observation. She was discharged on day 7.

She has not complained of right hip pain for the past 2 years since her discharge.

## Discussion

In 1871, Virchow^7^ used the term myxoma to describe a lesion that resembled the mucinous substance of the umbilical cord.

Intramuscular myxomas are slow-growing benign tumors; they can be painless or painful. The pain can be related to compression of surrounding structures.

Inflammatory markers can increase in the setting of myxomas, such as C-reactive protein, erythrocyte sedimentation rate, and interleukin-6.^8^ In the case of our patient, the pediatric infectious disease team attributed her elevated inflammatory markers, fever, and shoulder and hip pain to the possible systemic effect of the underlying intramuscular myxoma.

Most musculoskeletal myxomas are intramuscular (82%) in location, occurring most often in the thigh (51%), upper arm (9%), calf (7%), and buttock (7%).^2,9^

Intramuscular myxomas have been also reported in the paraspinal muscles of the back and cervical spine.^10,11,12,13^ The only pediatric intramuscular myxoma case reported in the literature is for a 22-month-old girl with an intramuscular myxoma in the posterior cervical triangle.^1^

The definite diagnosis of an intramuscular myxoma suspected on advanced imaging studies requires a pathologic examination, which can be obtained by an open, incisional, or excisional biopsy or with a core biopsy guided with advanced imaging. In the case of our patient, we chose a CT-guided core biopsy because of the deep location of the mass and to decrease the patient's morbidity.

Intramuscular myxomas are usually solitary, unless they are a part of the Mazabraud syndrome, which is characterized by the association of single or multiple intramuscular myxomas, with a monostotic or polyostotic form of fibrous dysplasia.^14^

The differential diagnosis of our patient's case included an infectious etiology, such as a primary abscess of the obturator internus muscle^15^; this was excluded by the solid nature of the mass, which was noted during the CT-guided biopsy and the negative cultures. Localized neurofibroma was another possibility; they tend to affect a younger age group than intramuscular myxoma (20 to 30 years old); they are usually painless but can sometimes cause pain.^2^

No specific laboratory test exists for intramuscular myxoma. Carbohydrate antigen 19-9, a tumor marker, has been reported to be correlated with intramuscular myxoma; however, it may also increase in multiple other malignant and benign conditions.^16^ Next-generation sequencing for determining GNAS mutation status on small core biopsies can facilitate the diagnosis of intramuscular myxoma and differentiate it from other tumors such as low-grade myxofibrosarcomas.^17,18^

## Conclusion

Pediatric obturator internus intramuscular myxomas are extremely rare; however, they can have a presentation that mimic a more serious condition such as hip septic arthritis, pelvic osteomyelitis, and pelvic abscess. They should be considered in the differential diagnosis in a pediatric patient presenting with hip pain and a pelvic mass.
